# Supplementary material for: A fast MR-thermometry method for quantitative assessment of temperature increase near an implanted wire
Source: PLoS One. 2021 May 13;16(5):e0250636. doi: 10.1371/journal.pone.0250636 (PMC8118538; doi:10.1371/journal.pone.0250636)
Supplement: Attachment — Submitted filename: Response to Reviewers.docx [file pone.0250636.s001.docx]

**Response to Reviewers**

We thank both reviewers for their overall positive evaluation of our work and suggestions for improving the manuscript. We understand that the main criticisms were:

- Clarify how calibration curves can be used to actually evaluate the temperature rise of any MRI sequence
- How can this technique be used practically

In our initial manuscript, we wrote that calibration curves can be used to predict maximal temperature rise for any other MRI sequence. The idea of these calibration curves was to provide quantitative estimate of maximal temperature rise near the tip of the wire as a function of the total energy (through the fits of maximal ΔT vs flip angle, B1+rms or Energy, providing β_1_, β_2_ and β_3_ coefficients). From these fits, one would then be able to estimate the temperature rise of any other MRI sequence based on energy of the sequence only. This idea was driven by the current safety approach in MRI, where SAR and B1+rms are commonly accepted quantities to assess safety, both being time-averaged values.

When reading comments from the reviewers (and particularly those from reviewer #2), we realized that this idea is in fact incorrect in the context of an implanted wire, since temperature rise effectively depends on the emitted power and duration (thus is energy dependent) of a given MRI sequence, but that the most relevant parameter is the power since it is linked to the temperature rise. As an example, 100 W applied during 10s will produce a much higher heating than 1 W applied during 1000 s, although energy is identical. We thus worked to solve this issue and to improve the manuscript by providing a method that takes into account the power emitted by the sequence instead of its cumulative energy or B_1+rms_.

We introduced in the revised manuscript a temperature model derived from previously published work on high intensity focused ultrasound. Using this approach, we can exploit the temperature curves obtained from the SAR module sequence in a different manner. First, we approximated the nearly punctual heating source (tip of the wire) as a Gaussian shape. This assumption offers the advantage of allowing to use an analytical function of temperature evolution at the hottest point, already referenced in the literature for HIFU heating (where a Gaussian function is considered as a realistic model).

Then, we fit temperature evolution for each flip angle of the SAR module with this function to derive two parameters (α and τ) linked to tissue characteristics (absorption and thermal diffusivity).

For a MRI sequence depositing a power P_0_ over a duration D, one can then simulate temperature evolution and predict maximal temperature rise, which is the relevant parameter for safety consideration regarding IEC standards. This semi-empirical model is presented together with an additional data set, comparing temperature rises measured by an optical fiber with those predicted by the model for three MRI sequences having different duration and emitted power. We show that the method is accurate for predicting the maximal temperature rise within 5% error.

We believe these amendments clarifies the focus of the paper and provide more evidence of the interest and potential of the proposed method. We also discuss the potential operating modes in perspective of future use.

We unfortunately could not implement an in vivo experiments to evaluate this method due to several practical constraints. However, we think this paper is a proof of concept and we hope this new manuscript will receive a positive evaluation.

We again thank both reviewers for their comments that helped us to improve the manuscript and avoid publishing incorrect conclusions.

**Reviewer #1:** This work presents a method to assess heating near implanted wires during an MRI scan due to RF deposition and induced currents. It uses fast measurements of temperature using PRFS MR Thermometry interleaved with an RF heating module. The method was shown to be precise and accurate in a variety of ex vivo heating scenarios and an in vivo no-heat scenario. Experiments and analysis are scientifically sound. I appreciated the inclusion of the limitations and comparison to existing methods in the discussion. Clarification of the scope, methods, and figures are needed. After these adjustments, I believe the article is suitable for publication.

Introduction was amended to clarify the scope. The method section now includes a processing technique to predict temperature evolution for other MRI sequence from temperature data obtained during the calibration phase. New results have been added to illustrate this technique using 3 different MRI sequences with different power and duration. The discussion has also been amended accordingly.

**Specific comments:**

**R1.1)** The title implies the work is for measurement near implanted wires, but the intro (page 5, line 99), methods (page 8 line 172), and others refer to "implants” or “devices." This terminology should be clarified throughout, as no evidence is presented to discuss how the work would be applicable to other types of implanted devices.

We modified the text to remove any ambiguity since the method is effectively intended to assess temperature increase near an implanted wire.

**R1.2)** The use case for the work should be made clear. How do you see the method being used in practice? Is the method intended to be used during real-time monitoring, as a one-time
phantom calibration that exists in look-up-table form for each wire type, or something else?
How would this be used safely at the beginning of each MRI session (page 19, line 384) without applying heating to the patient? Clarifying this in the abstract, introduction, and discussion will help clearly define the impact of the work.

We addressed this question in the introduction, discussion and conclusion of the revised manuscript. Objectives of the method is three fold: 1) to present a real time and precise temperature monitoring technique with adequate spatial and temporal resolutions for the targeted application, 2) to create a calibration data set by exploiting this sequence using interleaved heating module with frequent (1s update rate) temperature estimates, and 3) propose a model for predicting the maximal temperature increase for other MRI acquisition sequences from this calibration dataset, without requiring sophisticated modelling or using surrogates of temperature measurements.

This manuscript aims at presenting the proof of concept and potential optimizations are proposed in the discussion section to avoid inducing excessive heating (as pointed out by reviewer #2 also) in the patient.

We propose typical use cases in the conclusion of the manuscript.

**R1.3)** How much artifact was seen around the tips of the lead wires tested? Did it interfere with
maximum heat voxel selection/voxel near thermocouple selection? How would the method
perform if the lead wire were oriented differently with respect to B0, thus potentially having a
larger artifact?

We are aware that this could be a limitation of the imaging sequence. However, in each experiment presented in this manuscript, the implanted wire was systematically positioned vertically into the gel, thus orthogonally to B_0_. This is now indicated in the material and method section for each experiment. No visible artifact could be identified in the thermometry slice crossing the tip of the implanted wire when no fiber-optic probe was present. For the pacemaker lead a small reduction in signal intensity was observed in 4 pixels but without impacting significantly the thermometry precision (also indicated into the revised manuscript).

**R1.4)** Is the low-pass filtering necessary for generating the calibration curves, or would the original data be precise enough? Would the filter latency (page 12, line 246) be an issue for clinical implementation?

The low-pass filter was introduced to reduce noise on temperature images. Whether this is necessary or not is directly related to the standard deviation of the thermometry. In our implementation at 1.5T, with the selected coils on these gels with the selected spatial resolution and echo time, we decided to include it to reach good temperature precision. The latency induced by this filter is acceptable (3 seconds) since in case an important temperature increase is observed, the sequence can be immediately stopped, without creating risks for the patient. In 3 s, a temperature of 55°C (+18°C above body temperature) is necessary to reach the lethal thermal dose (taking the CEM43 metric). Other types of filters may be included in the thermometry pipeline such as Kalman filters (as already stated in the discussion section of the original manuscript). However, we do not consider the current implementation is an issue for clinical application.

A sentence has been added in the discussion section:

“However, despite the proposed implementation creates a latency of 3 s, a temperature increase of 18°C is necessary to reach a lethal thermal dose within this time scale (according to the CEM43 model) which is unlikely to occur with optimized clinical devices.”

**R1.5)** Figure 1 could be expanded to include which dynamics were averaged together. Add a label the thermometry module.

Done

**R1.6)** Add titles to all sub-parts of Figures 2 and 3, and 6a.

Done

**R1.7)** Scale bar in Fig 2A blends in, consider a different color and moving outside the phantom into the black area and adding the value to the image.

We changed the bar color and location to avoid such a blend.

**R1.8)** Figures 3c, 4b, 5, 6b need plot legends.

Done

**R1.9)** Page 6 line 127: How long was each dynamic (with and without interleaving the heating
module)?

Each dynamic was 1 sec long and maintained constant during the acquisition. When heating module is played, the remaining “empty” delay before the following acquisition of slices is filled with pulses (see figure 1 and M&M section for details)

“TR/TE = 1000/18 ms” was already indicated in the original manuscript

We kept the original text unchanged.

**R1.10)** Page 9 line 175: How was the voxel of interest chosen?

The sentence was amended as follow:

“For each acquisition, the temperature evolution in the same pixel was analyzed, selecting the pixel with the maximal temperature increase at the end of the energy deposition (acquisition #90) from the temperature data corresponding to the largest FA_HM_”

**R1.11)** Page 9 line 179: add scan parameters to statistics section

The following sentence was added. ” The same scan parameters as described in section MRI-thermometry sequence were used”

**R1.12)** Page 9 line 189: is this FA for the thermometry imaging module or the RF heating module?

This FA is the flip angle of the thermometry sequence, since here the heating module emitted no energy (FA_HM_=0°)

**R1.13)** Page 10 line 199: Was there any appreciable cooling during the 5 dynamics that were averaged? Since this depends on # of slices, how many slices were used in each of the experiments?

No cooling was observed during the 5 successive acquisitions (see temperature curves in Fig 4b). Three slices were used in each of the experiments (already indicated in the original manuscript). The text in the manuscript was not modified, but we added a mark in Fig 4b (FA_HM_=40°) showing the temporal window corresponding to this averaging.

This will help the reader to see that temperature can be considered nearly constant over this temporal window.

**R1.14)** Page 10 line 202: Explain how energy emitted was computed.

The sentence was modified as follow:

“ The µT ± σT temperature values were plotted as a function of the flip angle, B1+rms and energy emitted (i.e. sum of the energies of each individual RF pulse, including pulses for imaging and pulses of the heating module) “

**R1.15)** Page 16 lines 307-314 should be in the methods section.

We moved this section to Methods section.

**R1.16)** Page 18 line 373: What is the regulatory limit?

In the latest version of IEC 60601–2‐33, the so‐called fixed‐parameter option (FPO) was introduced for 1.5T systems (FPO:B), which specifically addresses the scanning of implant carriers and fixed limit value of $B_{1,rms}^{+}$ = 3.2 µT. In Table 1, this correspond to FA_HM_ of 70° .

The sentence was amended as follow:

“In the experiments with the copper wire, we observed an important temperature increase when the flip angle of the heating module was higher than 30°, although B_1+rms_ values provided by the scanner interface remained within regulatory limit (maximal B_1+rms_ of 3.2 µT, as indicated in the fixed‐parameter option of IEC 60601 –2‐33) for most of the experimental conditions (FA_HM_ up to 70 °, see Table 1).”

**Reviewer #2:** This manuscript describes a method to run an MRI pulse sequence with an added “Heating module” to induce heating in implanted wires. The pulse sequence is a single-shot EPI pulse sequence for PRF MR thermometry, previously described in multiple papers by the same group. If the sequence is run multiple times with different parameters for the “Heating module”, the flip angle, B1+, and Energy can be plotted as functions of the temperature increase measured (with PRF MR thermometry) at the tip of a wire/lead. This creates what the authors call calibration curves, and which they claim can then be used to be sure temperature increases stay below regulatory limits even when other pulse sequences are used. Experiments were performed in agar gel phantoms to investigate accuracy (as compared to fiber optic temperature measurements) and precision of the MR thermometry, and in one healthy volunteer to investigate precision in vivo in brain.

**R2.1** Over all the approach is interesting and probably worth investigating. It is however not clearly described in the manuscript how the authors envision the approach being used, and the reader has to “read between the lines” to really understand the point of the “calibration curves”. This should be made clearer and described more straightforward in the introduction.

We agree that this was unclear in the original manuscript. We have thoroughly modified the manuscript to explain this in the introduction, added a section in material and methods, include new results and discuss them in the revised manuscript.

We hope these modifications bring clarity and a more precise focus to the manuscript.

**R 2.2** Secondly, it’s not clear why the authors went through the trouble of doing all these experiments and stopped short of actually evaluating the method for its intended purpose. The whole point of getting the calibration curves are so you can predict how much heating other (more clinically relevant) pulse sequence will induce. So, when doing the phantom experiment with the fiber optic probe, why didn’t the authors derive the calibration curves and then used them to predict how much heating a set of clinically relevant pulse sequences would induce, and then compare to what the probe actually measured? Without this experiment, the paper will be of very limited impact as it is not clear if the described method will actually work as intended. In my opinion this experiment (at the very least in phantom or maybe better in ex vivo tissue, but ideally in vivo in an animal model) must be included before the manuscript can be published.

Thank you very much for this criticism of the original manuscript. We have amended the method to introduce a heating model and propose a method to compute temperature evolution of other sequences from calibration data set using our proposed MR-thermometry sequence. We included an additional experiment to evaluate this method and discuss these points. Unfortunately, we could not organize an in vivo experiment for various practical reasons. This is clearly stated in the revised manuscript and we believe this new version brings enough new material to be considered for publication as a proof of concept. In the discussion, we propose potential improvements of the method and conclude with potential use-case scenarios.

We hope all these improvements will convince the reviewer.

**R 2.3** When doing this experiment the maximum temperature rise when getting the calibration curves should ideally be kept below 6 °C as this is generally when thermal dose starts to accumulate in vivo (i.e., at 43 °C assuming 37 °C starting temperature). Without this experiment the authors can probably not make statements/claims such as “Calibration curves derived from temperature measurements under different RF exposure levels were fit to predict temperature increase for any MR-acquisition sequence”.

We totally agree with the reviewer. In the revised version of the manuscript we added a paragraph to the discussion section to address this comment.

**R2.4** Lastly, the orientation of leads/wires inside the bore can affect how much heating and artifacts are created, and it is not clear how well the single-shot EPI sequence handles this. This is another straight forward experiment to perform that would improve the readers excitement about the paper.

Each experiment presented here was performed with the wire tip positioned vertically into the gel (thus perpendicular to B0). This was thus the worst case configurations. Please see answer to R1.3 for more details.

Minor comments:

**R2.5** Abstract

“...compared to invasive fiber-optic measurements to assess precision…“ this would assess accuracy and not precision?

We corrected this in the text.

**R2.6** “In gel, as well as in the human brain, temperature measurements within ± 0.2 °C
certainty” Please reformulate this. I assume this is from the SD through time, so maybe say something like “the precision of the measurement was 0.2 °C…”. Also mention that this is after temporal filtering.

We reformulated the sentence as suggested

**R2.7** Ln 117: The Introduction discusses the importance of a large enough FOV – why was such a small FOV (only 12 cm) chosen? That’s not practical for anything but maybe imaging extremities – certainly too small for head and body imaging.

We initially chose a small FOV to concentrate on only a local heating at the wire and pacemaker lead tips. However, as depicted in the human volunteer, it is possible to adjust the FOV depending on the region of interest and thus, acquiring with a larger FOV. The meaning of the sequence was that FOV must be large enough to cover regions around the implanted wire, while being rapid, precise and spatially resolved.

The sentence was modified as follow: “In the context of measuring small temperature changes near devices, MRI-thermometry should be rapid with a sufficiently large spatial coverage around the implanted wire and provide a spatial resolution in the range of a few millimeters.”

**R2.8** Ln 118: How many slices were interleaved in the 1000 ms TR?

3 slices were acquired every second. This was already indicated in the original text.

**R2.9** Ln 124: Change KHz to kHz

Corrected

**R2.10** Ln 125: TR is used above – define when it’s first used (and define other parameters above, too).

Corrected

**R2.11** Ln 136: What algorithm was used for EPI ghost correction?

Reconstruction pipeline included EPI ghost correction using three central line of k-space. We refer the reader to the paper by Ozenne et al for implementation details (reference #29 in the manuscript).

**R 2.12** Ln 138: Please change “pixel size” to “pixel spacing” (the size doesn’t change with zero filling, but spacing does)

We proceeded to change

**R2.13** Ln 152-154: This sentence is not clear. Did it take 6 minutes before you could run the sequence again? Why was that?

On the MR scanner, it takes 6 minutes to reset the B1+rms value to zero. As we wanted to consider the B1+rms emitted by one sequence only we had to wait this delay. Acquiring before this delay would have result in a mix of B1+rms emitted by several sequences. Moreover, this 6 minute delay ensured proper cooling of the gel before the following experiment with a different FA_HM_.

The text was amended accordingly.

**R 2.14** Ln 171-172: When removing probes from gel phantoms an air-filled “track” is often left behind, resulting in susceptibility artifacts – did you observe this? Or did you use a new/separate phantom for this experiment?

A new phantom was use for each separate experiment to avoid the mentioned issue of susceptibility artifacts induced by air-filled track of previous experiments. This mentioned in the revised manuscript.

**R2.15** Ln 174: Doing 10 heatings in a single location can seem like quite a lot – did you somehow check/control that the heatings were repeatable, by, e.g., repeating the same heating at the beginning, middle and end?

After the last heating was performed (largest FA_HM_ value), a delay was observed for cooling down. Then another sequence with FA_HM_ of 30° was acquired again to ensure same value of maximal temperature increase was obtained as for the previous experiment performed with identical FA_HM_. This was the case, demonstrating repeatability of the heating.

The text was not modified, since we believe this does not bring major added value to the manuscript.

**R2.16** Ln 211-212: Please use same number of significant figures for all numbers

This was corrected in the text.

**R2.17** Ln 252: Suggest change “given” to “measured”

Done

**R2.18** Ln 306: Most (all?) of this paragraph seems to belong better in the Materials section. This whole experiment wasn’t mention in the Materials at all.

We moved this paragraph in the method section, as suggested.

**R2.19** Ln 339: Again, interpolation doesn’t change the size of the voxel, just the spacing. So, the voxel size is 6.3 mm3 both before and after zero filled interpolation.

We removed “before interpolation” in the text.

**R2.20** Ln 348-350: Changing the TR will change the duty cycle no matter if the heating is fast or slow, so why do you say “since the temperature evolution is relatively slow”? Even if it was fast the duty cycle would change with changing TR? Or do you mean something else?

Of course, changing the TR results in a different duty cycle. This sentence suggested that considering the relative slow evolution of the temperature, the update time of temperature measurement could be increased (eg ever 2 seconds). This increase in TR might be invested in acquiring more EPI slices to increase the spatial coverage of the temperature measurement, if desired. However, this sentence does not appear essential and we prefer to remove it if it is unclear.

**R2.21** Ln 354-356: This was, however, after temporal filtering. Please add if references 24 and 26 also used temporal filtering. If they didn’t, how did your unfiltered values compare to theirs?

Comparing temperature SD results with those from Refs 24 and 26 is not straightforward since it depends on several parameters such as the SNR, which is linked to the voxel size and receiver coil performance. Ehses et al used a PRF thermometry technique with a larger voxel size of 1.56x1.56x5mm (12.168 mm^3^ vs 6.1 mm3 in our study) with an update time of 3.9 s (versus 1 s in our implementation). They spatially averaged temperature data in 2 adjacent pixels that resulted in 0.5°C standard deviation. Gensler used a T1-based thermometry and reported a temperature standard deviation of 1.37°C. However, their update time was 6.4 s for an elementary voxel size of 26.5 mm^3^.

In both cases, a precise comparison of their achievements with the standard deviation reported with our method appears hardly feasible, except using the raw values provided in their studies (0.5°C and 1.37°C) and compare them to our 0.2°C after temporal filtering, without taking into account the different voxel dimensions and update times.

Thus, we prefer to keep the original text unchanged.

**R2.22** Ln 386-388: “In this objective, real-time MRI-thermometry as proposed here is of central interest to avoid creating excessive temperature rise during the calibration process.” Well, yes, but you need the MR thermometry to create the curves in the first place.

In this study, we first propose a fast MR-thermometry method. Then, this is used to create a calibration data set from which one can derive maximal temperature increase using a model to fit temperature curves of the calibration data set. We report an example of our method using a medical device where small heating was observed (<6°C).

As pointed out in the discussion section, the model allows to fit temperature data during heating and cooling. Thus optimization of the calibration process can be envisioned, with the idea of reducing the duration of the heating module (here 80 s). This paper is thus a proof of concept of the method with a lot of potential improvement in perspective of clinical use and will require further studies, including in vivo application.

Please see the discussion section that covers these points.

**R2.23** Of course, but having thermometry does not mean that you need to heat excessively. The use of thermometry in real-time avoids reaching excessive temperature (eg no larger than 43°C).

This is now discussed in the revised manuscript.

**R2.24** Ln 396: Do you mean Figure 2b?

Yes, thank you for having noticed.

**R2.25** References

Please check all references. They seem to contain months (and other things?) in French rather than English, etc.

Corrected

**R2.26** Figures

The Figures are overall fairly low quality (at least in the provided pdf), so it’s hard to see any details. Please include higher resolution figures.

This is due to the conversion process.
