# Supplementary material for: A fast MR-thermometry method for quantitative assessment of temperature increase near an implanted wire
Source: PLoS One. 2021 May 13;16(5):e0250636. doi: 10.1371/journal.pone.0250636 (PMC8118538; doi:10.1371/journal.pone.0250636)
Supplement: Attachment — Submitted filename: Response to Reviewers.docx [file pone.0250636.s002.docx]

**Answer to comments**

We thank both reviewers for their positive evaluation of the revised manuscript.

Please find below our point-by-point response to their comments.

**Reviewer #1:** The manuscript has been greatly improved this round. All previous comments have been addressed. A new set of analysis and figures have been added to address previous concerns around how the work is to be used/calibration curves. These were effective additions. I also appreciate the text that was added to the conclusion discussing how the work can be used. Given the substantial additions to the text and figures, all comments below relate to the added work. Once addressed I believe the article is suitable for publication.

**R1.1:** Figure 7 (and associated results/methods text) –

Report the goodness of fit: We reported the goodness of fit within the figure.

Add text justifying why a second order polynomial should be used. Based on the figure I don't think there is enough data to justify a second order polynomial:

Please see answer to comment R1.5 below

Add subfigure labels a and b to the figure itself: done

**R1.2:** Figure 8 –

Add units to the temperature isotherm lines (or indicate in caption): we added the unit (°C) in the subfigure label for fig b

Add subfigure labels a and b to the figure itself:

Done

**R1.3:** Page 9 ~line 200 and Page 12 ~line 260 (in tracked changes version) - indicate wire direction relative to scanner B0, not relative to the gel.

Done at both places.

**R1.4:** Page 12 (in tracked changes version) - Make sure all symbols used in eqn 1 are explicitly defined (I couldn't find T, t, alpha, and tau)

This is now detailed. There were also typo errors in the equation that are now corrected.

**R1.5:** Page 23 line 504 (in tracked changes version) - clarify whether the alpha and tau were allowed to vary in the data shown in the figures. I'm not convinced they should be allowed to vary unless you're seeing temperature rises above coagulations thresholds.

Alpha and tau were allowed to vary in the data shown in the figures. In the discussion section we already mentioned this explicitly and discussed that point:

“the two parameters resulting from the fit (here α and τ) are directly linked to tissue absorption and thermal diffusivity, and are thus not expected to vary with the emitted power. However, allowing α and τ to vary in our processing improved the fitting quality on each temperature curve of the calibration dataset. This was attributed to the relative small dimensions of the heating spot for low FA_HM_ values, where partial volume effect of the thermometry sequence may play a role, although an effort was made to provide high resolution temperature images with the proposed sequence. This semi-empirical model allowed to predict the maximal temperature increase for three other MRI sequences.”

Of course, alpha and tau may vary with coagulation but this is not the use case of the method since the plan is to create a moderate heating and use temperature curves to predict the maximal temperature rise for any other sequence.

Moreover, if you compare the values of alpha and tau derived from the fits for the two first experiments (FA_HM_ =10° and 20°) in Fig 7b and 7c, they appear different although there is no expected change in gel composition with temperature increases below 2°C (FA_HM_=10°) and 6°C (FA_HM_ = 20°) starting from room temperature (~20°C). Thus, we attributed these changes to partial volume effect of the thermometry sequence that is more pronounced for very low temperature increase, despite we tried to use a high resolution thermometry sequence.

The text was modified as follow:

“Whatever the model, indeed, the two parameters resulting from the fit (here α and τ) are directly linked to tissue absorption and thermal diffusivity, and are thus not expected to vary with the emitted power, at least for moderate temperature increases (i.e. remaining below the lethal thermal dose). However, parameters α and τ (Fig 7b and 7c) derived from the fit of temperature curves (Fig 7a) show a strong variation when varying FAHM from 10° to 20° and lower changes for higher FAHM (30° and 40°). For these reasons, we introduced a polynomial fitting function in Fig. 7b and 7c. Variation of α and τ as a function of the power was attributed to the relative small dimensions…”

The following sentence was removed:

“However, allowing α and τ to vary in our processing improved the fitting quality on each temperature curve of the calibration dataset.”

**Reviewer #2:** Thank you for answering my previous questions and updating the manuscript, which now is clearer and easier to follow. I just have a few minor suggestions on this latest version.

**R2.1:** Abstract, Purpose; Suggest adding that you use scans from one sequence to predict heating for other sequences.

The sentence now reads: “To propose a MR-thermometry method and associated data processing technique to predict the maximal RF-induced temperature increase near an implanted wire for any other MRI sequence”

**R2.2:** Ln 51: Is 0.5 °C maximum error, RMSE etc.? Please clarify.

Thank you for this remark. The difference between maximal predicted and measured temperature increases was around 5% for the three tested sequences.

Therefore, we corrected the sentence as follow:

“After successful fitting of temperature curves of the calibration data set, temperature rise predicted by the model was in good agreement (around 5% difference) with measured temperature by a fiber optic probe, for three other MRI sequences. “

**R2.3:** Ln 204-205: Please reformulate “The tip of the lead screwed into the myocardium was inserted…”

We reformulated the sentence as follow:

“The tip of the lead that is normally screwed into the myocardium was inserted into the gel while the other extremity was left in the air”

**R2.4:** Ln 225-227: What was the TR for these scans? Was it the same for all TEs (if not, compare the precision later on gets challenging)?

Repetition time was identical for each measurement and set to 1s. This is now indicated in the text.

**R2.5:** Ln 249-250: Does the Gaussian have the same width in all three dimensions (what was it?)? Or is it elongated like most focused ultrasound focal spots?

Yes, we assume a Gaussian source with isotropic dimensions. In our model, we do not need to explicitly provide a width for the Gaussian function, since this is included in the parameter “tau”. The idea here is that if you have a Gaussian source of heating, then you can use Eq 1 to fit the temperature curve vs time at the hottest point to derive alpha and tau (see ref 35 for details).

In order to avoid any confusion, we removed « as for high intensity focused ultrasound » from the sentence that now reads:”

“Considering that heating induced near an implanted wire is localized around its tip, we chose to approximate this heating source by a Gaussian function with isotropic dimensions.”

**R2.6:** Ln 350: This reviewer can’t seem to find any blue line (showing “baseline”)? Also, consider calling it “background” rather than “baseline”.

Effectively, we forgot to include these blue lines in the figure. Instead, we added a blue arrow (as in Figure 3) in Figure 4 to show the pixel selected for background temperature evolution. We also changed “baseline” into “background” as suggested.

**R2.7:** Ln 408-410: It’s a bit unclear what you mean with this sentence - simply that you can use the plot in 5c to estimate the maximum temperature rise?

What is meant here is that the method relying on time-averaged power deposition are irrelevant to predict the maximal temperature rise.

The sentence has been modified as follow to clarify this point:

“On the contrary, using the calibration curves (namely coefficient β_3_) from Fig 5c to estimate the maximal temperature increase from the total energy of each sequence leads to 2.78°C, 2.96°C and 2.42°C, respectively. Such an approach is thus irrelevant for predicting maximal temperature rise in the context of an implanted wire.”

**R2.8:** Ln 436: “(1 s refresh rate in our setting)” please add “with a 5 s temporal foot print”. This is important, as you had to shift the temporal curve to align it with the probe measurements. Hence, it is not optimally suited for real-time applications (similar to using a sliding window reconstruction for undersampled k-space data).

We added “ with a 3 s temporal footprint”, not 5 s, since this is the time shift we needed to align MR-temperature curve and probe measurements.

**R2.9:** Ln 448: Change “FA ≥ 20°” to “FA_HM_ ≥ 20°”, right?

Changed

**R2.10:** Ln 472: This doesn’t quite tell the full story, right? This assumes a “step function” going straight to 18 °C and holding it there for 3 s. In reality you will start accumulating dose as soon as your increase is 6 °C so with the fairly slow heatings shown in Fig 4 you’ll have a substantial dose before getting to 18 °C.

These numbers were introduced in the revised manuscript to illustrate that having a 3 s latency is not considered problematic, since a substantial heating (18°C) is required to reach a lethal thermal dose within this time scale. In the discussion section, we already explained that the sequence may be used with shorter duration of the heating module, which will also reduce the risk of accumulating excessive thermal dose. Altogether, we believe that this 3 s latency is not an issue. In order to simplify the text, we propose to modify the sentence as follow:

“Despite the proposed implementation creates a latency of 3 s, the risk of reaching a lethal thermal dose within this time scale (according to the CEM43 (40) model) is unlikely to occur with optimized clinical devices.”

**R2.11:** Figure 8: Both the CINE and TSE predict the heating and start of cooling pretty well, but starts to deviate substantially at the end of the cooling period – can the authors speculate why this is?

This deviation can result from uncertainty in estimation of alpha and tau parameters that are used to simulate the heating. In this case, deviation may accumulate and become more visible at the end of the cooling. However, as stated in the discussion section, the relevant parameter from the IEC is the maximal temperature rise, which is predicted within 5% error with our method. Since this point is already indicated in the previous revised manuscript, we prefer to keep the text unchanged.
